# Maternal Fecal Microbes Contribute to Shaping the Early Life Assembly of the Intestinal Microbiota of Co-inhabiting Yak and Cattle Calves

**DOI:** 10.3389/fmicb.2022.916735

**Published:** 2022-06-06

**Authors:** Jianbo Zhang, Zeyi Liang, Renqing Ding Kao, Jianlin Han, Mei Du, Anum Ali Ahmad, Shengyi Wang, Ghasem Hosseini Salekdeh, Ruijun Long, Ping Yan, Xuezhi Ding

**Affiliations:** ^1^Key Laboratory of Yak Breeding Engineering, Lanzhou Institute of Husbandry and Pharmaceutical Sciences, Chinese Academy of Agricultural Sciences, Lanzhou, China; ^2^Key Laboratory of Veterinary Pharmaceutical Development, Ministry of Agricultural and Rural Affairs, Lanzhou Institute of Husbandry and Pharmaceutical Sciences, Chinese Academy of Agricultural Sciences, Lanzhou, China; ^3^Gannan Institute of Animal Husbandry Science, Hezuo, China; ^4^Livestock Genetics Program, International Livestock Research Institute, Nairobi, Kenya; ^5^Chinese Academy of Agricultural Sciences (CAAS) and International Livestock Research Institute (ILRI) Joint Laboratory on Livestock and Forage Genetic Resources, Institute of Animal Science, CAAS, Beijing, China; ^6^School of Life Sciences, Lanzhou University, Lanzhou, China; ^7^Department of Systems Biology, Agricultural Biotechnology Research Institute of Iran, Agricultural Research, Education, and Extension Organization, Karaj, Iran

**Keywords:** intestinal microbial colonization, intestinal microbial succession, host-microbiome interaction, maternal microbial transmission, natural grazing yak

## Abstract

The Qinghai-Tibetan Plateau offers one of the most extreme environments for yaks (*Bos grunniens*). Although the genetic adaptability of yak and rumen metagenomes is increasingly understood, the relative contribution of host genetics and maternal symbiotic microbes throughout early intestinal microbial successions in yaks remains elusive. In this study, we assessed the intestinal microbiota succession of co-inhabiting yak and cattle (*Bos taurus*) calves at different weeks after birth as well as the modes of transmission of maternal symbiotic microbes (i.e., rumen fluid, feces, oral cavity, and breast skin) to their calves’ intestinal microbiota colonization. We found that the fecal microbiota of yak and cattle calves after birth was dominated by members of the families *Ruminococcaceae*, *Bacteroidaceae*, and *Lachnospiraceae*. The Source Tracker model revealed that maternal fecal microbes played an important role (the average contribution was about 80%) in the intestinal microbial colonization of yak and cattle calves at different weeks after birth. Unlike cattle calves, there was no significant difference in the fecal microbiota composition of yak calves between 5 and 9 weeks after birth (Wilcoxon test, *P* > 0.05), indicating that yak may adapt to its natural extreme environment to stabilize its intestinal microbiota composition. Additionally, our results also find that the intestinal microbial composition of yak and cattle calves, with age, gradually tend to become similar, and the differences between species gradually decrease. The findings of this study are vital for developing strategies to manipulate the intestinal microbiota in grazing yaks and cattle for better growth and performance on the Qinghai-Tibetan Plateau.

## Introduction

The animal gastrointestinal tract is inhabited by a diverse microbial community, which influences a wide range of host metabolic processes, immune system, central nervous system development, and even behavior (Diaz Heijtz et al., 2011; Hooper et al., 2012; Tremaroli and Backhed, 2012). This complex community of microbes must be reassembled each generation since before birth infants lack a gastrointestinal tract microbiota (Smits et al., 2017). There is increasing evidence that microbial colonization is a complex process influenced by a two-way interaction between the host and microbial community as well as a variety of external factors, such as neonatal delivery, maternal and environmental microbiota, diet, parenting behavior, and early use of antibiotics (Dominguez-Bello et al., 2010; Rothschild et al., 2018; Liu et al., 2019; Chen et al., 2020; Furman et al., 2020). Studies have revealed the essential roles of environmental and maternal microbes in the establishment of newborn microbiota, which is likely critical for protecting the newborn from pathogens when its immune system is immature (Round and Mazmanian, 2009; Gensollen et al., 2016; Gomez de Aguero et al., 2016). However, young ruminants are considered functionally monogastric at birth with underdeveloped forestomach system, including the rumen, reticulum, and omasum (Heinrichs, 2005). Dietary nutrients obtained from hindgut is likely an important source of energy for ruminants throughout all stages of their development, while hindgut fermentation could be of an elevated importance to the calves during their first days of life before the rumen is fully developed (Castro et al., 2016b). In contrast to that of rumen microbial ecosystem, the fundamental role of intestinal microbiota and its contribution to ruminant health and production in neonatal calves are less well understood. Thus, knowledge about the possible sources of early intestinal microbiota and their colonization may help to explore the functional interaction between host metabolism and intestinal microbiota.

The yak (*Bos grunniens*), a herbivore exclusively inhabiting in the Qinghai-Tibetan Plateau (QTP) and adjacent mountainous regions, evolutionarily diverged from cattle (*Bos taurus*) about 4.4 to 5.3 million years ago (Gu et al., 2007). It has been found that yak are superior to cattle in feeding and grazing behavior (Ding et al., 2008), digestive organ structure (Shao et al., 2010; Guan et al., 2017), nitrogen use efficiency (Zhou et al., 2018), low rumen methane emission (Ding et al., 2010), and interseason energy utilization efficiency (Ahmad et al., 2020; Yang et al., 2020). A whole genome sequencing study has identified potentially functional genes related to the unique adaptation of yak to severe hypoxia condition (Qiu et al., 2012). A recent study argued that yak adaptation mechanisms to harsh environment and long-term nutritional stress on the QTP are related to the enrichment of key genes for volatile fatty acid (VFA) fermentation pathway in rumen microbiome while methanogenesis pathway are enriched in cattle (Zhang et al., 2016). Furthermore, rumen microbial compositions change during the growth of yak from neonatal (7 days) to adult (12 years) stages, especially the bacterial and archaeal groups are more sensitive in response to development stages compared to the two eukaryotic microbial groups (Guo et al., 2020). Notably, most of the above-mentioned studies focused on the structure, function, and succession of the rumen microbial community in yak, but there is limited knowledge about the development of intestinal microbiota between yak calves (YB) and cattle calves (CB) in the same habitat before weaning.

Some studies have shown that maternal microbes quickly colonize offspring gastrointestinal tracts after parturition through birth canal, skin contact or breast feeding and shape the onset of an intestinal immune system and its future development (Bi et al., 2019; Klein-Jobstl et al., 2019; Lima et al., 2019; Fan et al., 2020). Traditionally, YB are mainly fed by maternal nursing under natural grazing condition during the pre-weaning period, and we hypothesized that this period helps the calf to acquire more maternal microbes, such as fecal, saliva, and skin microbes. Therefore, we collected fecal samples in YB and CB from 1 to 9 weeks after birth, as well as rumen fluid, feces, oral cavity, and breast skin swabs of their mothers at 1 week post parturition for 16S rRNA gene amplicon sequencing. The information obtained in this study is vital for the future development of strategies to manipulate the intestinal microbiota in grazing yak and cattle for better growth and performance in the harsh QTP ecosystem.

## Materials and Methods

### Animal Experiments and Sampling

Both YB and CB were born naturally, fed with milk by maternal suckling, and grazed on the same native pasture (without concentrate supplementation) in Yangnuo Specialized Yak Breeding Cooperative (34°43′19.66″N, 102°28′49.51″E) at Xiahe county of Gannan Tibetan Autonomous Prefecture, Gansu Province, China. All the animals involved in this experiment were from the same herd and they all grazed together in an alpine meadow on the QTP, where the average altitude is 3,300 m and the average annual temperature is 4°C. There were abundant natural alpine meadow herbage and water resource, and the animals freely drank water from the local river or the snow meltwater. All the animals were grazed from 7 a.m. to 6 p.m., and the samples were collected before the morning grazing. All animals included in this study were healthy during our sampling period and received no recorded therapeutic or prophylactic antibiotic treatment. Both YB and CB were born naturally, fed with milk by maternal suckling, and grazed on the same native pasture. In addition, the calves are usually weaned and managed separately by herders more than 2 months after birth in our study.

**FIGURE 1:**
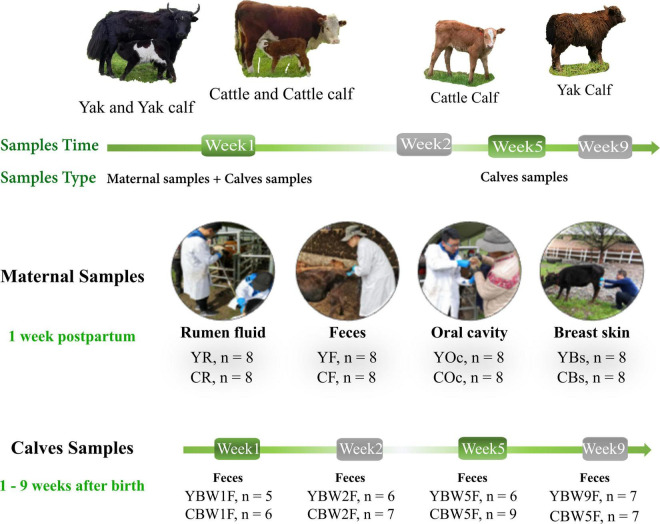
Intestinal microbial source and succession analysis of yak and cattle calves on the same pasture at different weeks during pre-weaning. From June to August 2019, fecal samples were collected from yak calves (YB) and cattle calves (CB) at weeks 1 (W1), 2 (W2), 5 (W5), and 9 (W9) after birth by inserting a gloved finger into the anus of calf to stimulate defecation. Rumen fluid (R), feces (F), oral cavity (Oc), and breast skin (Bs) swabs from their mothers were also sampled at 1 week post parturition. YBW1F, YBW2F, YBW5F, and YBW9F represent fecal samples from yak calves at weeks 1, 2, 5, and 9 after birth; CBW1F, CBW2F, CBW5F, and CBW9F represent fecal samples from cattle calves at weeks 1, 2, 5, and 9 after birth.

From June to August 2019, fecal samples were collected from YB and CB at weeks 1 (W1), 2 (W2), 5 (W5), and 9 (W9) after birth by inserting a gloved finger into the anus of the calf to stimulate defecation. Rumen fluid (R), feces (F), oral cavity (Oc), and breast skin (Bs) swabs from their mothers were also sampled 1 week post parturition. An overview of the experimental design is shown in Figure 1. Initially, we selected a total of 20 pregnant animals—yak (*n* = 10) and cattle (*n* = 10)—but their exact gestation period was not known. When the calf was born, fecal samples from both YB and CB aged 1 week (YBW1F: *n* = 5; CBW1F: *n* = 6), 2 weeks (YBW2F: *n* = 6; CBW2F: *n* = 7), 5 weeks (YBW5F: *n* = 6; CBW5F: *n* = 9), and 9 weeks (YBW9F: *n* = 7; CBW9F: *n* = 7) were repeatedly sampled before their morning grazing, of which six YB and seven CB were consistently sampled from the first to the ninth week. Unfortunately, some calf feces could not be sampled due to uncontrollable factors, such as calf death. At the same time, fecal samples (YF: *n* = 8; CF: *n* = 8) of their mothers were obtained from the rectum using sterile gloves and lubricants. Rumen fluid sample (YR: *n* = 8, CR: *n* = 8; average 50 ml/per animal) from the mothers was collected prior to the morning grazing *via* a perforated stainless-steel stomach tube connected to a suction pump. The first 20 ml rumen fluid was discarded to avoid contamination with saliva. Samples of the oral cavity (YOc: *n* = 8; COc: *n* = 8) and breast skin (YBs: *n* = 8; CBs: *n* = 8) were collected by swabbing the mouth and breasts with a sterile cotton swab (Universal Transport Medium for Bacterium, Beijing, China), immediately placing it inside a sampling tube, and immersing in a protective solution (1.5 ml). All samples were immediately frozen in liquid nitrogen, transported to the laboratory and stored at −80°C until DNA extraction.

### DNA Extraction and Illumina Sequencing of 16S rRNA Genes

The samples collected included calf feces at four time points (*n* = 53), maternal feces (*n* = 16), maternal rumen fluid (*n* = 16), maternal oral cavity swabs (*n* = 16), and breast skin swabs (*n* = 16). All 117 samples were subjected to the same sample preparation and DNA isolation procedure. Sample preparation was done to DNA isolation to optimize microbial loads for 16S rRNA gene PCR amplification. The swab samples were oscillated for 15 s to remove bacteria from the swabs and centrifuged at 12,000 × *g* for 10 min at 4°C. The cotton swab was then carefully removed and the centrifugation step was repeated. The pellets obtained was used for DNA isolation. Rumen fluid and fecal samples did not require sample preparation; genomic DNA was extracted using 220 mg of thawed homogenized sample. Total genomic DNA from all the samples (*n* = 117) was extracted using hexadecyl trimethyl ammonium bromide (CTAB) method (Honoré-Bouakline et al., 2003). DNA concentration and purity were monitored using 1% agarose gels. DNA was diluted to a final concentration of 1 ng/μl using sterile distilled water. The 16S rRNA gene V4 region was PCR-amplified using the following specific primers: 515F 5′-GTGCCAGCMGCCGCGGTAA-3′ and 806R 5′-GGACTACHVGGGTWTCTAAT-3′ with barcodes (Caporaso et al., 2011). All PCR reactions were carried out in 30 μL reactions with 15 μL of Phusion^®^ High-Fidelity PCR Master Mix (New England Biolabs), 0.2 μM of forward and reverse primers, and approximately 10 ng template DNA. Thermal cycling consisted of initial denaturation at 98°C for 1 min, followed by 30 cycles of denaturation at 98°C for 10 s, annealing at 50°C for 30 s, and elongation at 72°C for 30 s, and finished by a final extension at 72°C for 5 min. Amplicons were purified with Qiagen Gel Extraction Kit (Qiagen, Germany). Sequencing libraries were generated using TruSeq^®^ DNA PCR-Free Sample Preparation Kit (Illumina, United States) following manufacturer’s recommendations and index codes were added. The library quality was assessed on the Qubit@ 2.0 Fluorometer (Thermo Scientific) and Agilent Bioanalyzer 2100 system. At last, the library was sequenced on an Illumina NovaSeq platform (Novogene, Tianjin, China) and 250 bp paired-end reads (PE 250) were generated.

### Bioinformatic and Statistical Analysis

The paired-end reads were assigned to samples based on their unique barcode (Fu et al., 2021a) and then data were imported to QIIME2 (version: 2020.8.0) pipeline for further analysis (Bolyen et al., 2019). Briefly, (i) primers were removed by “qiime cutadapt trim-paired” (–p-minimum-length 200); (ii) sequences were denoised using dada2 algorithm (“qiime dada2 denoise-paired”) to obtain feature sequences (amplicon sequence variant, ASVs) and table (–p-trim-left-f 15 –p-trim-left-r 20 –p-trunc-len-f 0 –p-trunc-len-r 0 –p-n-threads 6) (Callahan et al., 2016), features with frequency less than 4 were removed; (iii) the sequences from SILVA database (release 132) (Quast et al., 2013) were extracted using specific primers for V4 region to train Naive Bayes classifier for taxonomy assignment using “qiime feature-classifier classify-sklearn,” ASVs assigned to mitochondria and chloroplast were excluded from feature table. Alpha diversity of fecal microbiota was characterized by Chao1 and Shannon diversity indices using “qiime diversity alpha” command line. Statistical comparison of the alpha diversity indices between group levels was performed using the Wilcoxon rank-sum test. Non-metric multidimensional scaling (NMDS) plots of the Bray–Curtis metric were calculated with square root transformed data and visualized in R (vegan package Version 2.5-4) (Oksanen et al., 2013). Permutational multivariate analysis of variance (PERMANOVA) was used to examine the differences of feces microbial communities between calves and their mothers among different age groups. The linear discriminant analysis (LDA) effect size (LEfSe) algorithm was used for differential analysis to identify significantly different taxa (Segata et al., 2011). In addition, we used SourceTracker2 (Knights et al., 2011), a Bayesian community-level microbial source-tracking tool, to estimate the proportion of sequences in the calf feces microbiota that originated from different parts of their mother’s body. SourceTracker2 was run with default parameters using non-rarefied data; each calf intestinal microbial communities were designated as a sink, and all maternal sample types were designated as sources. In order to identify and sort the genera which contributed the most to the differences in fecal microbial community between YB and CB at different weeks after birth, a similarity percentage analysis (SIMPER) was performed using PAST (Version 3.1.7) (Hammer et al., 2001). All genera below a defined threshold (90%) of cumulative contributions were declared as specialized genera. In this analysis, SIMPER determined the specialized genera in the calf feces microbiota based on the changes in cumulative contributions to explain differences among developmental stages and species groups. Based on the 16S rRNA sequences, the function of the intestinal microbial communities in calf was predicted using Phylogenetic Investigation of Communities by Reconstruction of Unobserved States (PICRUSt) (Langille et al., 2013), and the functional prediction was assigned according to the standard method (Agrawal et al., 2019). The predicted functional contents were summarized at Kyoto Encyclopedia of Genes and Genomes (KEGG) pathway hierarchy levels 2 and 3 for interpretation and subsequent analysis. The two-sided Welch’s *t*-test and Benjamini–Hochberg FDR correction were used in two-group analysis.

## Results

### Richness and Diversity of Calves and Maternal Microbiota

**FIGURE 2:**
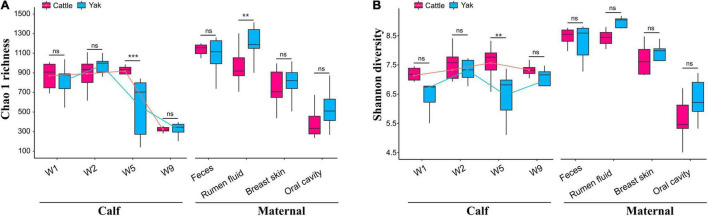
Richness and diversity of the calf and maternal microbiota. **(A)** The Chao1 species richness and **(B)** Shannon diversity indexes of microbial communities in the samples of yak and cattle calf feces, and their mothers. W1, W2, W5, and W9 represent fecal samples from calves at weeks 1, 2, 5, and 9 after birth. The lines in the box plot show the trend of microbial community diversity in the feces of yak and cattle calves at different weeks after birth. Statistical differences were analyzed using the Wilcoxon tests: ***P < 0.001, **P < 0.01, and ns denotes a non-significant difference.

We found differences between maternal and calf fecal microbiota, wherein maternal samples (F, R, Bs, and Oc) harbored higher species richness and more diverse microbiota than calf fecal samples at different weeks after birth (Wilcoxon test, *P* < 0.05; Supplementary Table 1). In the fecal microbiota of 2-week-old YB and CB, Chao1 species richness was not significantly different between the two species (Wilcoxon test, YBW1F vs. CBW1F, *P* = 0.792, *Q* = 0.840; YBW2F vs. CBW2F, *P* = 0.366, *Q* = 0.439). Interestingly, at 5 weeks of age, the decrease in Chao1 species richness and Shannon diversity indices of fecal microbiota in YB was significantly different from that of CB (Chao1, YBW5F vs. CBW5F, *P* = 0.008, *Q* = 0.016; Shannon, YBW5F vs. CBW5F, *P* = 0.026; *Q* = 0.050; Supplementary Table 1). Compared to CB, there was no significant difference in the species richness and diversity of fecal microbiota in YB at the fifth and ninth weeks after birth (Chao1, *P* = 0.366, *Q* = 0.439; Shannon, *P* = 0.366, *Q* = 0.439; Supplementary Table 1). At 9 weeks after birth, the difference in Chao1 species richness and Shannon diversity indices of fecal microbiota between YB and CB was not statistically significant (Chao1, YBW9F vs. CBW9F, *P* = 0.608, *Q* = 0.686; Shannon, YBW9F vs. CBW9F, *P* = 0.128, *Q* = 0.186; Figures 2A,B and Supplementary Table 1), whereas Chao1 species richness of the fecal microbiota between pre-weaned calves and their mothers was significantly different (YBW9F vs. YF: *P* < 0.001, *Q* = 0.002, CBW9F vs. CF: *P* < 0.001, *Q* = 0.002; Supplementary Table 1). There were no significant differences in the microbial diversity of the fecal, oral cavity, and breast skin of adult yak and cattle (Chao1, *P* > 0.05; Shannon, *P* > 0.05; Figures 2A,B and Supplementary Table 1). However, the species richness of rumen microbial community was higher in yak compared to cattle (Chao1, *P* = 0.038, *Q* = 0.069; Shannon, *P* = 0.007, *Q* = 0.015).

### Early Life Calf Intestinal Microbial Community Structure

**FIGURE 3:**
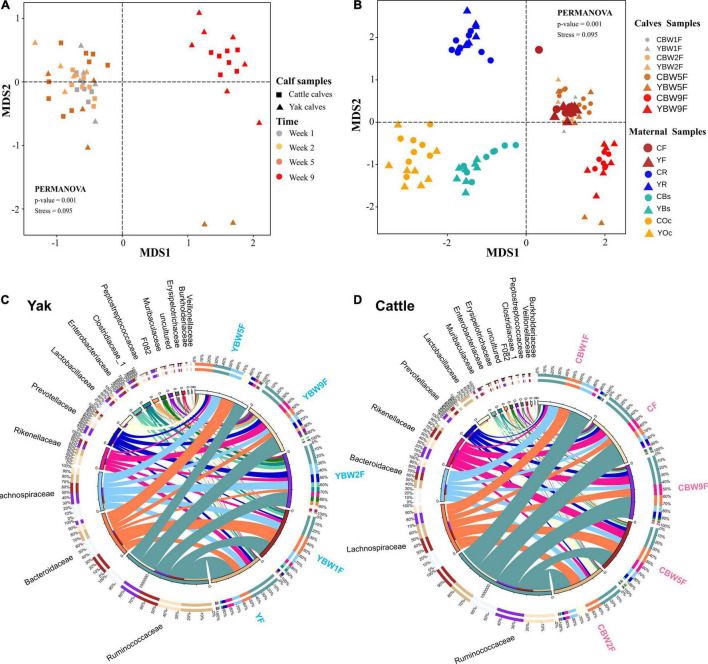
Diversity and composition of microbial communities in calf and maternal samples. **(A)** NMDS plot based on Bray–Curtis distance shows the differences between yak and cattle calves in different weeks after birth, and the differences between groups were analyzed by PERMANOVA. **(B)** NMDS plot based on Bray–Curtis distance shows the differences between calves and their mothers’ samples at different time points. A greater distance between two points infers a lower similarity, whereas similar ASVs cluster together. YR, YF, YBs, and YOc represent rumen fluid, feces, breast skin, and oral cavity samples from yak at 1 week post parturition; CR, CF, CBs, and COc represent rumen fluid, feces, breast skin, and oral cavity samples from cattle at 1 week post parturition. Circos diagram shows the composition of fecal microbiota at the family level (top 15) of yak **(C)** and cattle calves **(D)** in different weeks after birth, respectively. The length of the bars on the outer ring and the numbers on the inner ring represent the percentage of relative abundance of genera detected in each sample and the number of sequences in each sample, respectively. The bands with different colors show the source of each sequence affiliated with different clusters.

We examined how the maternal microbial community influences the intestinal microbiota structure of co-inhabiting YB and CB during the pre-weaning period (Figure 3). NMDS was performed on maternal microbiota (fecal, rumen fluid, oral cavity, and breast skin) and calf fecal microbiota at 1, 2, 5, and 9 weeks of life using Bray–Curtis distance matrices (Figures 3A,B). The NMDS plot showed clear separations between calf fecal and maternal samples in both species (yak and cattle). Maternal oral cavity and breast skin samples clustered together, all maternal and calf fecal samples formed a separate cluster, while maternal rumen samples stood alone (Figure 3B). There was no significant difference in the fecal samples of YB and CB at 1 week after birth (PERMANOVA, *P* = 0.389; Supplementary Table 2). However, the fecal microbial communities between YB and CB were significantly different at 2, 5, and 9 weeks of age (PERMANOVA, *P* < 0.05; Supplementary Table 2). Of the 42 bacterial phyla identified, six dominated calf fecal microbiota (average cumulative abundance = 99.1%), of which Firmicutes, Bacteroidetes, and Proteobacteria were also common in the intestinal microbiota of adult bovines and calves (Supplementary Figure 1). However, Proteobacteria was the dominant phylum in the maternal oral cavity and breast skin, followed by Firmicutes. At the family level, we found that *Ruminococcaceae*, *Lachnospiraceae*, and *Bacteroidaceae* were prevalent in the fecal samples of both pre-weaning YB and CB (Figures 3C,D). Moreover, *Bacteroides*, *Ruminococcaceae_UCG-005*, *Rikenellaceae_RC9_gut_group*, and *Lactobacillus* were the dominant genus in the fecal microbiota of both YB and CB after birth.

### Predictive Source Tracking of Calf Intestinal Microbial Communities During the Pre-weaning Period

**FIGURE 4:**
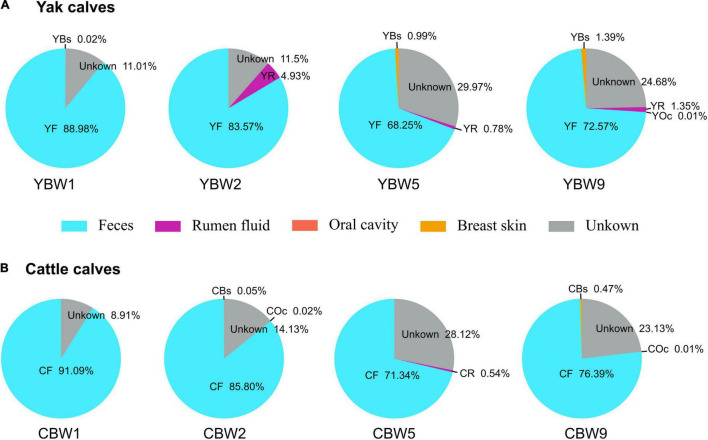
Source Tracking model of the maternal sources in early calf intestinal community assembly. We used SourceTracker2 to estimate the proportion of sequences in the intestinal microbiota of both yak **(A)** and cattle calves **(B)** at different weeks after birth that originated from their maternal microbial communities. YBW1F, YBW2F, YBW5F, and YBW9F represent fecal samples from yak calves at weeks 1, 2, 5, and 9 after birth; CBW1F, CBW2F, CBW5F, and CBW9F represent fecal samples from cattle calves at weeks 1, 2, 5, and 9 after birth. YR, YF, YBs, and YOc represent rumen fluid, feces, breast skin and oral cavity samples from yak at 1 week post parturition; CR, CF, CBs, and COc represent rumen fluid, feces, breast skin, and oral cavity samples from cattle at 1 week post parturition.

Bayesian community-level source tracking (Matamoros et al., 2013) was used to investigate the contribution of maternal microbiota to the intestinal microbial community assembly of both YB and CB. The Source Tracker model also revealed the roles of the microbial communities of different regions (rumen fluid, feces, oral cavity, and breast skin) of yak and cattle in shaping the intestinal microbiota of their calves (Figure 4 and Supplementary Figures 2, 3). On average, 88.98% of the fecal microbiota of YB at 1 week of age was derived from maternal feces source while less than 0.02% was from the maternal breast skin and 11.01% from unknown sources (Figure 4A). Similarly, 91.09% of the fecal microbiota of CB originated from maternal feces source while less than 8.91% was from unknown sources (Figure 4B). On average, both YB and CB at 2 weeks of age still harbored more than 80% of the maternally originated fecal microbial communities (Figures 4A,B). In addition, the proportions of microbiota from the maternal rumen fluid, breast skin, and other unknown sources in calf intestinal microbiota tended to increase with age (Figures 4A,B). Thus, the results suggest that maternal fecal microbiome may play an important role in the early colonization of calf intestinal microbiome.

### Influence of Maternal Fecal Microbiota on Early Calf Intestinal Microbial Succession

**FIGURE 5:**
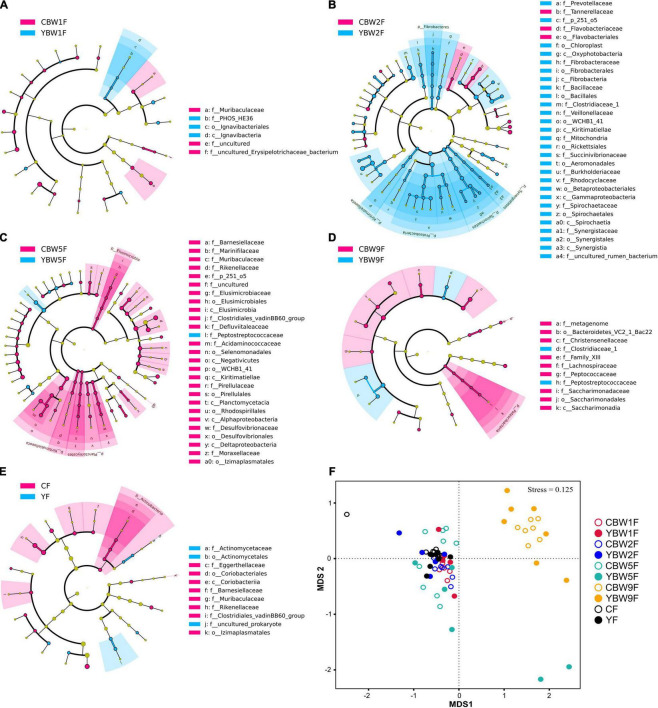
Linear discriminant analysis effect size analysis of the intestinal microbial community structure of yak and cattle calves at different weeks after birth. The cladograms indicate the phylogenetic distribution of the intestinal microbiota of yak and cattle calves at 1 week of age **(A)**, 2 weeks of age **(B)**, 5 weeks of age **(C)**, 9 weeks of age **(D)**, and their mothers at 1 weeks postpartum **(E)** using the linear discriminant analysis (LDA) effect size (LEfSe) method. Differences are represented by treatment colors (blue indicates yak, pink indicates cattle, and yellow non-significant). Circles represent taxonomic ranks from domain to species from inside to out layers. The LDA cut-off score is 2. Letters in front of ASVs represent taxonomic levels (p, phylum; c, class; o, order; and f, family). **(F)** The gut microbial community structures across calf and maternal samples. Non-metric multidimensional scaling (NMDS) ordination based on Bray–Curtis distances among sample types are plotted based on ASV abundances in the calf and maternal fecal samples. YF and CF represent fecal samples from yak and cattle during the first week of postpartum, respectively.

To further determine the influence of maternal fecal microbial community on calf early intestinal microbial succession, LEfSe and NMDS analysis were performed to determine whether calf intestinal microbial community structure changed with age (Figure 5 and Supplementary Figure 4). At 1 week of age, *Butyricimonas*, *Barnesiella*, and *Terrisporobacter* were the most abundant genus colonizing yak calf intestinal, whereas that of CB was more abundant in *Faecalibacterium*, *Ruminococcus_torques_group*, *Ruminococcus_gauvreauii_group* (Supplementary Figure 4A). In 2-week-old calves, Proteobacteria, Fibrobacteres, and Spirochaetes were more abundant in the fecal microbiota of YB at 2 weeks after birth than in that of CB, but the relative abundance of Elusimicrobia, Planctomycetes, and Kiritimatiellaeota in the fecal microbiota of CB at 5 weeks after birth was higher than YB (at phylum level, Figure 5C). In addition, we found that the fecal microbiota of YB at 9 weeks was more abundant in *Peptostreptococcaceae*, and *Clostridiaceae_1*, whereas that of CB was more abundant in *Lachnospiraceae*, *Christensenellaceae*, and *Peptococcaceae* (at the family level, Figure 5D). Meanwhile, the fecal microbiome composition of adult yak and cattle were similar, differing in only a few microbes (Figure 5E). The similarity cluster analysis based on NMDS (stress = 0.125) showed good agreement with the results of the LEfSe analysis (Figure 5F and Supplementary Table 3). With the development of the intestinal tract, there was no significant difference in the fecal microbiota composition between yak and cattle (PERMANOVA, YF vs. CF, *P* = 0.333; Supplementary Table 3). Therefore, our results further suggest that the differences in the fecal microbiota between YB and CB decrease with age, making their intestinal microbiota more similar.

### Establishment of Specialized Microbial Communities in the Intestine of Yak and Cattle Calves Before Weaning

**FIGURE 6:**
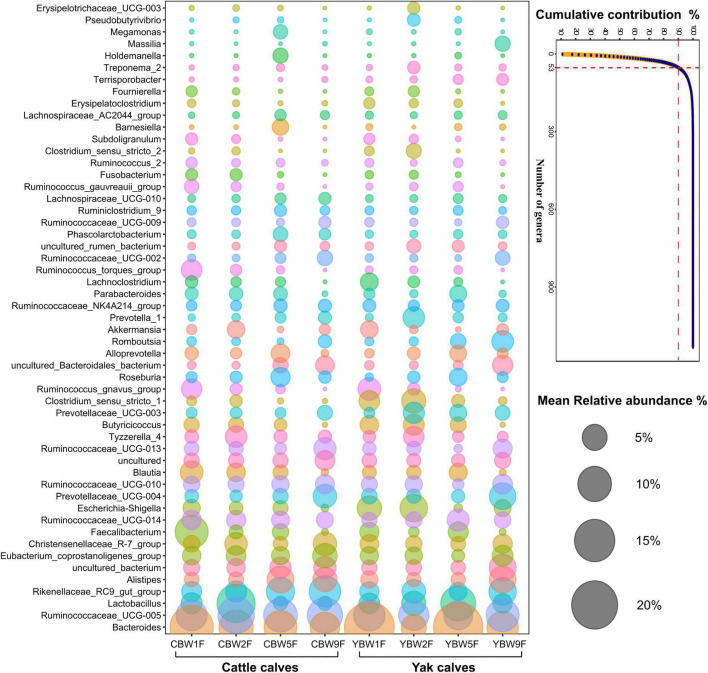
Similarity percentage analysis (SIMPER). The line graph shows the result of the SIMPER analysis performed with the PAST program, where all genera under a defined threshold (90%) of the cumulative contribution are declared as specialized genera. The number of genera indicates the accumulation of types of microbial communities at the genus level. Bubble-plot represents specialized microbial communities in the intestines of yak and cattle calves before weaning, while the size of the bubble represents the relative abundance of the respective genus.

To identify the genera that predominantly contribute to differences the intestinal microbiota between YB and CB at different weeks after birth, at specialized community was defined based on similarity percentages breakdown (SIMPER) analysis. We found that only 4.68% of the overall microbial community in fecal samples, that is, 53 of 1,132 genera with the highest variabilities in their relative abundances were responsible for 90% of the dissimilarities between the communities in YB and CB at different ages (Figure 6). There were some unique microbial communities in the intestines of YB and CB at different weeks after birth. Consistent with the fecal microbial composition of YB and CB at different weeks of life (Supplementary Figures 1B,C), we found that the relative abundance of *Bacteroides*, *Ruminococcaceae_UCG-005*, *Lactobacillus*, and *Rikenellaceae_RC9_gut_group* were the highest (Figure 6). In 1-week-old calves, the relative abundance of *Bacteroides* and *Lachnoclostridium* in the fecal of YB were higher than that of CB, while the relative abundances of *Faecalibacterium* in the fecal of CB was higher than that in YB. However, the relative abundances of *Lactobacillus* and *Alistipes* in the fecal of CB at 2 weeks of life was higher than that in YB. In 5-week-old calves, the relative abundance of *Bacteroides* and *Lactobacillus* in the fecal of YB were higher than that of cattle calve. In 9-week-old calves, the relative abundances of specialized microbial communities in the intestine of YB were similar to that of CB.

### Predicted Function of the Intestinal Microbiota in Pre-weaning Calves

**FIGURE 7:**
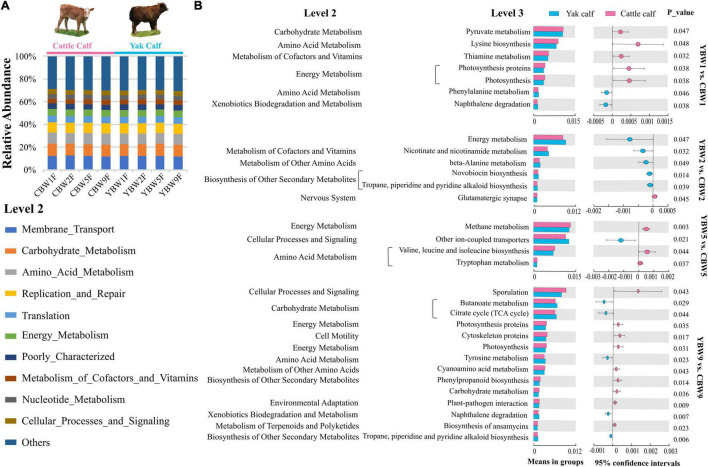
Prediction of potential function of the fecal microbial community in calf using PICRUSt. **(A)** Relative abundances of level 2 KEGG pathways are described by the age of calves within different species (different weeks after birth) in circular bar plots. **(B)** The comparison of the function of the fecal microbiota of yak and cattle calves at different weeks after birth based on KEGG level 3 annotation. The statistical significance of the comparison between groups was examined with *t*-test, and *P* < 0.05 indicates significant differences.

To assess the metabolic potential of the intestinal microbiota, PICRUSt-based functional prediction revealed differences in microbial functions in the intestinal microbial communities between YB and CB at different weeks after birth. These manifested into a difference in carbohydrate metabolism, amino acid metabolism, energy metabolism, metabolism of cofactors and vitamins, and another secondary metabolite biosynthesis (Figure 7A). One week birth, there was no significant difference in the fecal microbial function of YB and CB, such as pyruvate metabolism (*t*-test, *P* = 0.047; FDR, *Q* = 0.351), lysine biosynthesis (*t*-test, *P* = 0.048; FDR, *Q* = 0.351), and thiamine metabolism (*t*-test, *P* = 0.032; FDR, *Q* = 0.351) (Figure 7B and Supplementary Table 4). However, 2 weeks after birth, the fecal microbial function of YB and CB were focused on energy metabolism (*t*-test, *P* = 0.047; FDR, *Q* = 0.694), nicotinate and nicotinamide metabolism (*t*-test, *P* = 0.032; FDR, *Q* = 0.694), and beta-alanine metabolism (*t*-test, *P* = 0.049; FDR, *Q* = 0.694). It was found that the fecal microbiota function in YB and CB at 5 weeks after birth were focused on methane metabolism compared to other metabolic pathways (Figure 7B and Supplementary Table 4). Notably, we found that the differences in the fecal microbial function of YB and CB at 9 weeks after birth were tended to gradually increase, but the statistical differences were not significant, such as butanoate metabolism (*t*-test, *P* = 0.029; FDR, *Q* = 0.136), the citrate cycle (*t*-test, *P* = 0.044; FDR, *Q* = 0.136), and carbohydrate metabolism (*t*-test, *P* = 0.036; FDR, *Q* = 0.136).

## Discussion

Some studies have shown that microbial fermentation in the hindgut may be responsible for up to 30% of cellulose and hemicellulose degradation in ruminants (Hoover, 1978; Gressley et al., 2011). Understanding how microbial communities develop is essential for predicting and directing their future states (Sprockett et al., 2018; Cavicchioli et al., 2019). Although patterns of microbial colonization in the pre-functioning rumen have been the subject of several recent investigations (Jami et al., 2013; Malmuthuge et al., 2013; Cersosimo et al., 2019), there are noticeably fewer published reports concerning the hindgut microbiota of young ruminants (Alipour et al., 2018). Hence, the objective of our study was to explore the composition and possible sources of the intestinal bacterial community in YB and CB after birth and identify changes in the intestinal microbiota during normal development. Most studies have mainly investigated Holstein-Friesian calves (Castro et al., 2016a; Liu et al., 2019), whereas we analyzed the intestinal microbiota development in YB, the animals predominantly used for dairy and meat in the QTP region. The QTP, as the “third polar” of the world, offers an extreme and variable environment with high altitude, hypoxia, long cold season, strong ultraviolet radiation, and limited forage resources, making it ideal for studying high-altitude adaptation and species radiation (Long et al., 2008). However, yaks have adapted to the hard natural environment on the Tibetan plateau through long-term evolution and natural selection.

### Maternal Influence on the Assembly of Calf Intestinal Microbiota During Early Development

There is increasing evidence of microbial colonization being a complex process influenced by the interaction between host and microbes as well as a variety of external factors (Dominguez-Bello et al., 2010; Maslowski and Mackay, 2011; Rothschild et al., 2018; Liu et al., 2019; Chen et al., 2020). Compared with barn feeding, the source of gastrointestinal tracts microbes in young animals reared under natural grazing conditions may be more complex and diverse (Cui et al., 2020). It has recently been reported that calf fecal microbiota during the first 2 days of life is similar to its mother’s vaginal microbiota, suggesting that some of the fecal microbiota may be derived from the birth canal during parturition (Klein-Jobstl et al., 2019). However, we found that maternal fecal microbiota is critical in the rapid establishment and colonization of the intestinal microbiota in calves at different weeks after birth, and this effect persists until weaning or even longer. Several studies on other species have suggested that the intestinal microbiota is transferred from mother to offspring through social interaction, shared environment, and diet (Tung et al., 2015; Moeller et al., 2016; Rothschild et al., 2018; Chen et al., 2020). In addition, studies have found that initially colonized microbiota in the neonatal gastrointestinal tracts has origins in the maternal vagina (Backhed et al., 2015; Klein-Jobstl et al., 2019), breast milk (Derakhshani et al., 2018), and fecal microbes (Dominguez-Bello et al., 2016; Deng et al., 2019). Unfortunately, in our study, cow vaginal samples were not collected to assess their role in calf intestinal microbiome colonization as yak and cattle graze naturally and the birth of their calves is difficult to determine. Parental care may add diverse parental microbes, such as skin microbes, to newborns during the early stages of the intestinal microbial development (Colston and Jackson, 2016; Dominguez-Bello et al., 2016). This is essential for the establishment of the microbiome and helps resist pathogens when the immune system is not well developed in newborns (Round and Mazmanian, 2009; Gensollen et al., 2016; Gomez de Aguero et al., 2016). Hence, the advantage of direct inoculation of microbiota *via* physical contact with the dam deserves to be investigated further. In contrast to previous studies (Phillips, 2004; Abecia et al., 2014), our study focused on the influence of maternal microbiota on the intestinal microbial community of calves at different weeks after birth. We demonstrated the importance of maternal fecal microbiota on the rapid colonization of the intestinal microbiota of calves at different weeks after birth.

Previous studies have found that feeding strategies (natural or artificial) prior to weaning affected rumen microbial colonization and rumen development (Abecia et al., 2014). We found that YB and CB during the pre-weaning period are raised by maternal grazing and nursing, which is beneficial to the survival and growth of calves in extreme environments. Besides, it has been also reported that the establishment of intestinal microbiota within the first 7 weeks of life is associated with calf health and growth (neonatal diarrhea, pneumonia, and weight gain), and colonization by enteric pathogens might be responsible for dysbiosis in the intestinal microbiota of neonatal diarrhea (Oikonomou et al., 2013). Recent studies have shown that fecal microbiota transplantation (FMT) helps treat intestinal diseases and establish a healthy intestinal microbiota (Borody and Khoruts, 2011; Aroniadis and Brandt, 2013; Smits et al., 2013). At the same time, a recent FMT trial in ruminants suggests that FMT is capable of ameliorating diarrhea in pre-weaning calves with dysbiosis, and that FMT may play a role in improving growth performance (Kim et al., 2021). The findings of these studies provide an important scientific basis for the further use of maternal fecal microbiota to improve calves’ health. Considering previous and our findings, we suggest that calves should live with their mothers for a period of time after birth as this may help calves to quickly establish stable intestinal microbiota and thus reduce the economic losses caused by pre-weaning calf diarrhea.

### Dynamic Colonization of Intestinal Microbiota Structure in Calves Before Weaning

Studies of genetic adaptation, a central focus of evolutionary biology, most often focus on the host’s genome but rarely on the co-evolved microbiota. Previous studies on rumen microbial diversity in yak found that the yak rumen microbiota possesses more efficient fiber-degrading and energy-harvesting abilities than that of cattle from low altitudes (Zhang et al., 2016). However, little is known about the mechanism by which intestinal microbiota develops during the pre-weaning period and mature in grazing yaks. We found that except at the 5 weeks after birth, there was no significant difference in the fecal microbial richness and diversity between YB and CB. Additionally, we found that the fecal microbial diversity of YB and CB increased gradually at different weeks after birth, but the fecal microbial species richness of calves decreased at the 5 weeks after birth. This is in contrast to a recent study by Liu et al. (2019) who found that the fecal microbial diversity and species richness of dairy calves from 1 to 10 weeks after birth continued to increase and the fecal resistome of dairy cattle was associated with diet during nursing. Therefore, we postulate that it may be due to maternal grazing and nursing in YB and CB in the pre-weaning period, which was beneficial for the rapid colonization of intestinal microbiota of calves after birth compared to dairy calves separated from mothers after birth. However, a recent study revealed that there is no significant difference in the bacterial abundance of the rumen bacterial community in YB in the first 2 months after birth, but the rumen microbial community of adult yaks is significantly different from that of calves (Guo et al., 2020). Similarly, we also found significant differences in the Chao1 species richness in the fecal microbiota of pre-weaned calves and their mothers, suggesting that the intestinal microbial community become more abundant and diverse with age. In addition, we also found that the rumen fluid and fecal microbiota of yak and cattle comprised mainly of Firmicutes and Bacteroidetes, and Firmicutes comprised more than 55% of the fecal microbiota of YB and CB at different weeks after birth. Other studies have also found that the intestinal microbiota of calves comprised mainly of Firmicutes, Proteobacteria, and Bacteroidetes (Oikonomou et al., 2013; Malmuthuge et al., 2014; Yeoman et al., 2018). In this study, *Ruminococcaceae*, *Lachnospiraceae*, *Bacteroidaceae*, and *Lactobacillaceae* were the most abundant families in the fecal samples of YB and CB before weaning. *Ruminococcaceae* and *Lachnospiraceae* are butyrate-producing bacteria, indicating that they provide energy to the host by promoting the degradation of plant fibers (Ogata et al., 2019; Palevich et al., 2019). Studies have shown that *Bacteroidaceae* can degrade different plant polysaccharides, but studies on humans revealed that they do not respond effectively to fiber supplementation (El Kaoutari et al., 2013). *Bacteroides* members are generally considered for their ability to digest a broad range of plant cell-wall polysaccharides (Martens et al., 2011; El Kaoutari et al., 2013). We found that *Bacteroides* were the dominant genus in the intestinal microbiota of YB and CB in different weeks after birth. Moreover, members of the genus *Lactobacillus*, known as probiotics, produce lactic acid as the major end-product of carbohydrate metabolism and play an important role in nutrition, growth, and protection from infection (Drissi et al., 2014).

Previous studies have shown that original colonizers (*Streptococcus* and *Enterococcus*) (Malmuthuge et al., 2015) utilize oxygen available in the intestine and create an anaerobic environment for strict anaerobic intestinal residents (such as *Bifidobacteria* and *Bacteroides*) (Backhed et al., 2015). Similarly, our data showed that *Bacteroides* and *Lactobacillus* was abundant in the fecal microbiota in YB than in CB at 1 week after birth. It has been reported that the administration of *Bifidobacterium* and *Lactobacillus* to newborn calves during the first week of life increased weight gain and feed conversion ratio and decreased the incidence of diarrhea (Abe et al., 1995). There was no significant difference in Chao1 species richness and Shannon diversity indices of fecal microbiota between YB and CB at 2 weeks. However, our data suggest that the relative abundance of some fecal microbial communities, such as the main cellulolytic and hemicellulolytic bacteria (*Fibrobacter, Prevotellaceae, Clostridium*, and *Eubacterium*), amylolytic bacteria (*Streptococcus* and *Ruminobacter*), proteolytic bacteria (*Acidaminococcus* and *Lachnospira*), and saccharolytic bacteria (*Succinivibrio, Lactobacillus*, and *Bifidobacterium*) was significantly higher in YB than in CB. Therefore, we postulate that these microbial communities in the yak intestines can effectively degrade and utilize fibrous plant materials to provide nutrients for the host, making them better able to adapt to the extreme environment of the QTP.

Diet is one of the main factors that influence the composition of intestinal microbiota; therefore, an earlier forage can promote the stability and healthy development of the intestinal microbiota of neonatal calves (Phillips, 2004; Vieira et al., 2012; Jami et al., 2013; Rey et al., 2014). In our study, YB and CB living with their mothers in natural grazing conditions may have provided calves with more opportunities to learn to forage early and promote the development of the intestinal microbiota. This is consistent with other studies that show that calves reared in the presence of older companions exhibit more frequent and longer visits to the feeder, which was hypothesized to be the consequence of social learning (De Paula Vieira et al., 2012). In addition, inherited host-associated factors, such as genotype, gender, and immune status, might function as selective filters in the assembly of the intestinal microbiota (Benson et al., 2010; Bolnick et al., 2014). However, we found that the difference in the fecal microbiota of YB and CB at early stages gradually disappeared with age. We postulate that this may be because yak and cattle share the same habitat and diet structure, resulting in similar intestinal microbiota in adulthood. Similarly, a new study found that environment and host species identity shape the intestinal microbial diversity in sympatric herbivorous mammals (Fu et al., 2021b).

Intestinal microbes in early life are important for many aspects of animal immune (Sekirov et al., 2010), metabolic (Tremaroli and Backhed, 2012), and neurobehavioral traits (Montiel-Castro et al., 2013). Microbial amino acid metabolism, carbohydrate metabolism and energy metabolism are crucial in the hindgut and provide energy to the host (McNeil, 1984). We found that the function of calf fecal microbiota during the first 2 weeks of life was mainly focused on carbohydrate and amino acid metabolism, such as pyruvate metabolism, energy metabolism, and beta-alanine metabolism; therefore, we hypothesized that the early colonization of intestinal microbiota may provide more energy and amino acids to the calves to promote their growth. Notably, it was found that fecal microbiota function in methane metabolism in CB was significantly higher than that in YB at 5 weeks of age. Both low methane emissions and high VFA production in high-altitude mammals (yak) than in low-altitude mammals (cattle) have been observed before (Zhang et al., 2016). In addition, the observed temporal variations in the predicted fecal microbial functions of calves at 9 weeks after birth suggested that potential changes in carbohydrate metabolism of the calf intestinal microbiota may be related to changes in the structure of the host diet (milk to forage). However, we found several deficiencies in our study, such as the small number of experimental animals and sampling dates, and the lack of phenotypic observations and environmental samples. Although functional prediction using the 16S rRNA genome may provide preliminary information for studies on the intestinal microbial functions in calves at different weeks after birth, the detailed function of the intestinal microbiota needs to be further determined by techniques such as metagenomics, metatranscriptomics, and metabolomics.

## Conclusion

In this study, we investigated the effect of maternal microbiota on the succession of intestinal microbiota in YB and CB inhabiting the same natural pasture before weaning. We found that host species factors may play an important role in the composition of the fecal microbiota in YB and CB at different weeks after birth; however, this effect gradually disappeared with age. We also found that the diversity of fecal microbiota of YB after the fifth week of life compared to that of CB was relatively stable, suggesting a survival characteristic of yak to better adapt to the extreme environment on the QTP through natural selection and evolution. It is worth noting that, in our study, the intestinal microbiota of the calves raised by their mothers is established mainly through maternal transmission through the feces. However, this study only studied the origin and colonization of intestinal microbiota of YB and CB before weaning, and the differences of their intestinal microbiota structure and function after weaning should be further studied.

## Data Availability Statement

The datasets presented in this study can be found in online repositories. The names of the repository/repositories and accession number(s) can be found in the article/Supplementary Material.

## Ethics Statement

All experimental procedures in this study were approved by the Animal Care and Utilization Committee of Lanzhou Institute of Husbandry and Pharmaceutical Sciences of Chinese Academy of Agricultural Sciences (SYXK-2019-0012). Written informed consent was obtained from the owners for the participation of their animals in this study. Written informed consent was obtained from the individual(s) for the publication of any potentially identifiable images or data included in this article.

## Author Contributions

JZ, PY, and XD designed the study. JZ, ZL, RD, MD, AA, and SW collected the samples. JZ, ZL, RD, and MD performed the bioinformatics and statistical analyses. JH, SW, GS, RL, PY, and XD guided the data analysis and revised the manuscript. JZ, ZL, and XD interpreted the data and wrote the manuscript. All authors read and approved the final manuscript.

## Conflict of Interest

The authors declare that the research was conducted in the absence of any commercial or financial relationships that could be construed as a potential conflict of interest.

## Publisher’s Note

All claims expressed in this article are solely those of the authors and do not necessarily represent those of their affiliated organizations, or those of the publisher, the editors and the reviewers. Any product that may be evaluated in this article, or claim that may be made by its manufacturer, is not guaranteed or endorsed by the publisher.
