# Supplementary material for: Maternal Fecal Microbes Contribute to Shaping the Early Life Assembly of the Intestinal Microbiota of Co-inhabiting Yak and Cattle Calves
Source: Front Microbiol. 2022 Jun 6;13:916735. doi: 10.3389/fmicb.2022.916735 (PMC9208665; doi:10.3389/fmicb.2022.916735)
Supplement: Supplementary file 1 [file Data_Sheet_1.zip › Datasheet 1.docx]

**Maternal fecal microbes contribute to shaping the early life assembly of the intestinal microbiota of co-inhabiting yak and cattle calves**

Jianbo Zhang^1, 2†^, Zeyi Liang^1†^, Renqing Ding Kao^3^, Jianlin Han^4,5^, Mei Du^1^, Anum Ali Ahmad^1,6^, Shengyi Wang^2^, Ghasem Hosseini Salekdeh^7^, Ruijun Long^6^, Ping Yan^1*^, Xuezhi Ding^1, 2*^

1. Key Laboratory of Yak Breeding Engineering, Lanzhou Institute of Husbandry and Pharmaceutical Sciences, Chinese Academy of Agricultural Sciences, Lanzhou 730050, China.
2. Key Laboratory of Veterinary Pharmaceutical Development, Ministry of Agricultural and Rural Affairs, Lanzhou Institute of Husbandry and Pharmaceutical Sciences, Chinese Academy of Agricultural Sciences, Lanzhou 730050, China.
3. Gannan Institute of Animal Husbandry Science, Hezuo 747000, China.
4. Livestock Genetics Program, International Livestock Research Institute (ILRI), Nairobi 00100, Kenya.
5. CAAS-ILRI Joint Laboratory on Livestock and Forage Genetic Resources, Institute of Animal Science, Chinese Academy of Agricultural Sciences (CAAS), Beijing 100193, China.
6. School of Life Sciences, Lanzhou University, Lanzhou 730020, China.
7. Department of Systems Biology, Agricultural Biotechnology Research Institute of Iran, Agricultural Research, Education, and Extension Organization, Karaj, Iran.

**Running Title:** Succession and source of gut microbiota of yak

***Corresponding author:**

Xuezhi Ding (PhD)

E-mail: [dingxuezhi@caas.cn](mailto:dingxuezhi@caas.cn)

Ping Yan (PhD)

E-mail: [pingyanlz@163.com](mailto:pingyanlz@163.com)

^†^These authors have contributed equally to this study.

**Supporting information**

**Figures**


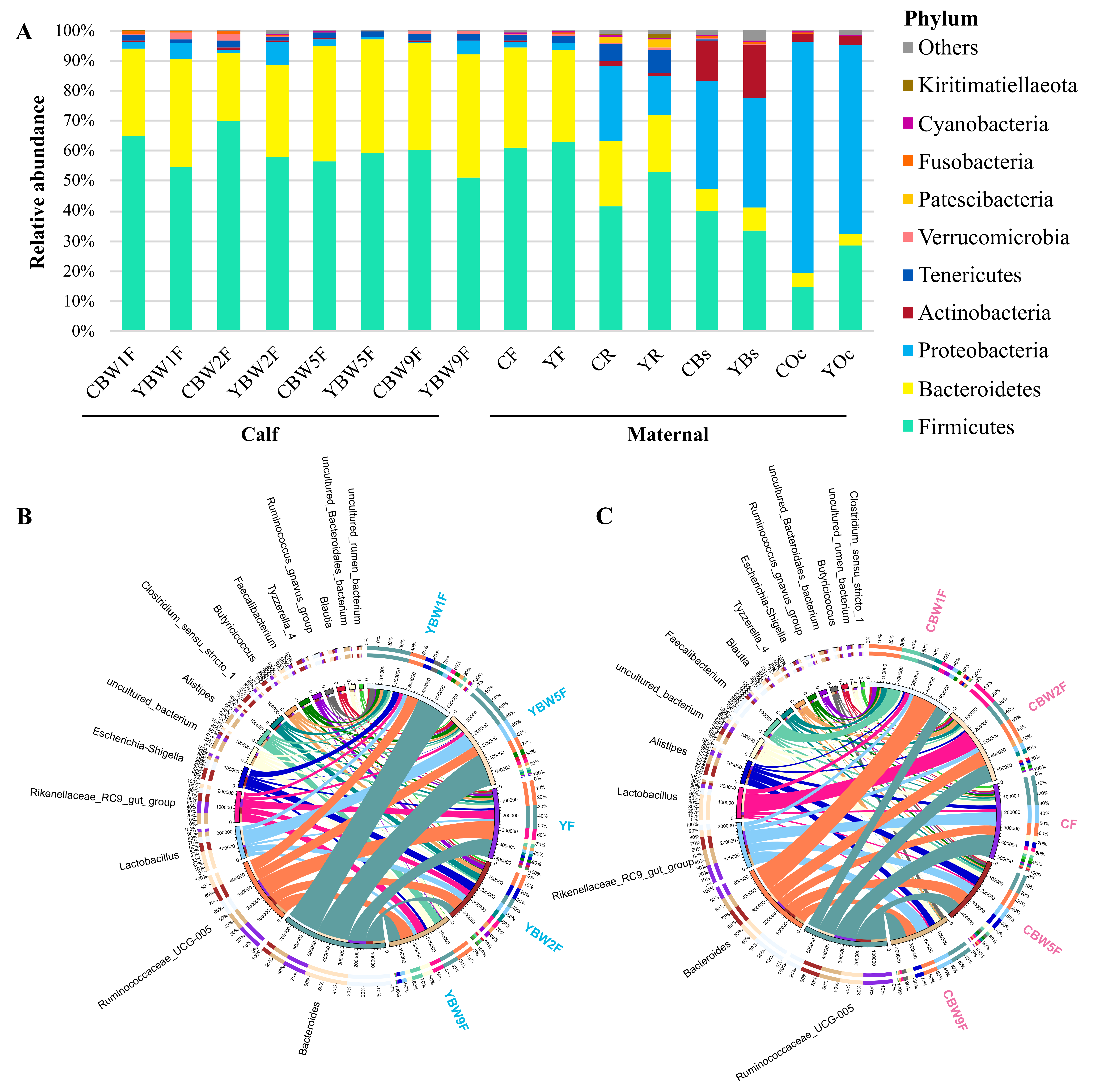


1. Composition of microbial communities in calf and maternal samples. (A) The average relative abundances of the most prevalent bacterial phyla in each sample type are plotted for samples from the maternal microbiota and calf feces. Circos diagram shows the composition of fecal microbiota at the genus level (Top15) of yak (B) and cattle calves (C) in different weeks after birth, respectively.


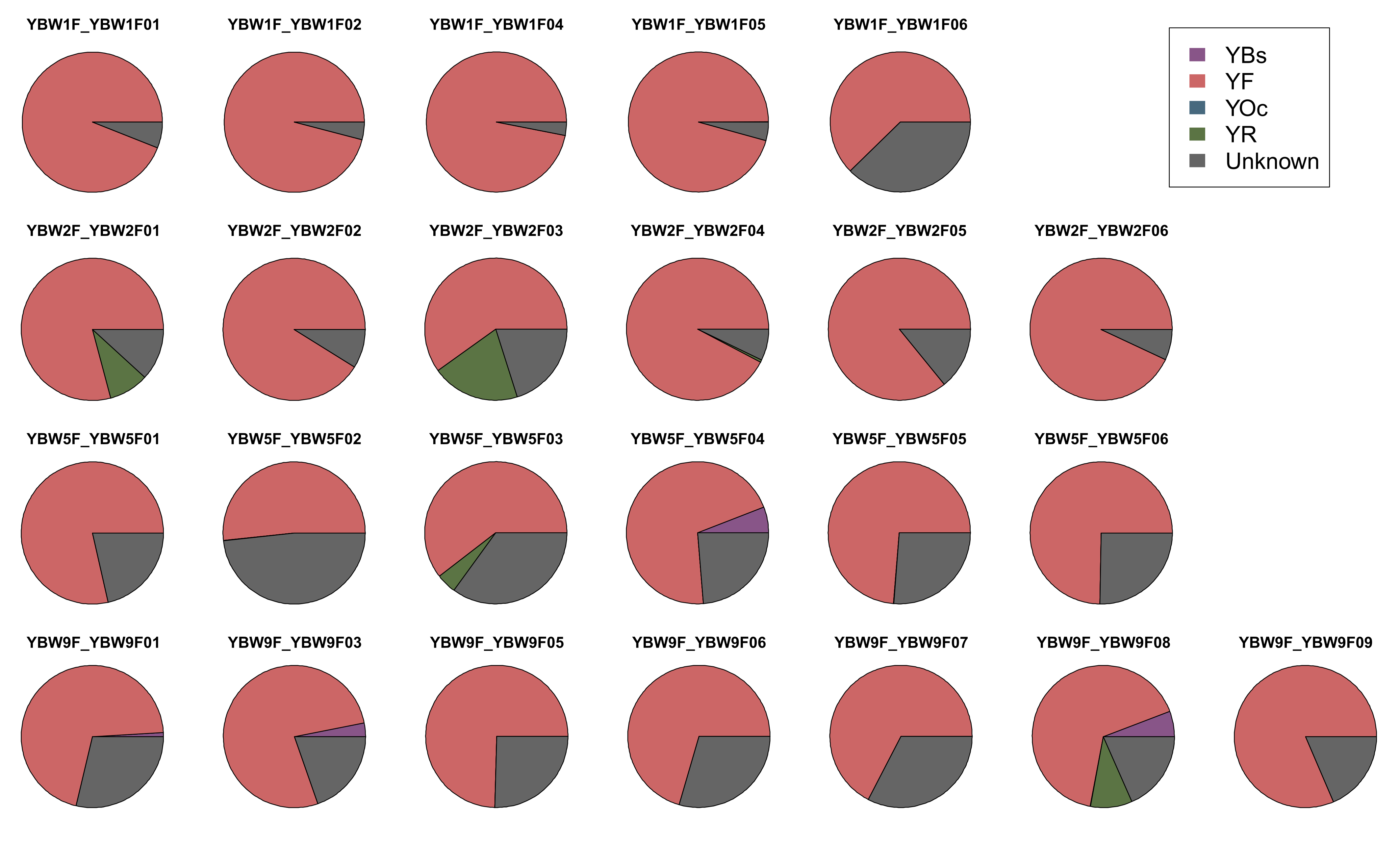


1. Contribution of maternal microbiota to intestinal microbiota of yak calves at different weeks after birth


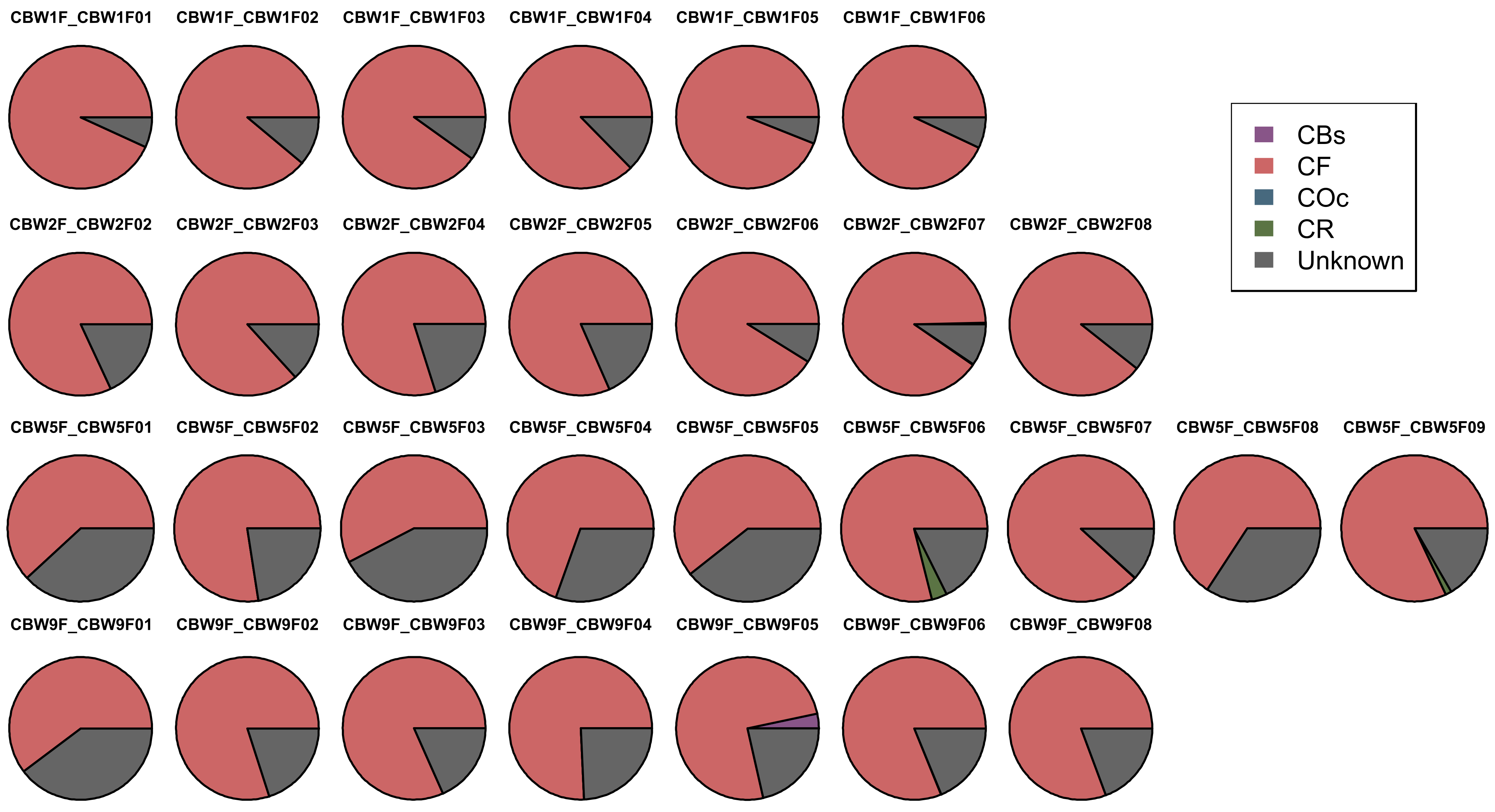


1. Contribution of maternal microbiota to intestinal microbiota of cattle calves at different weeks after birth.


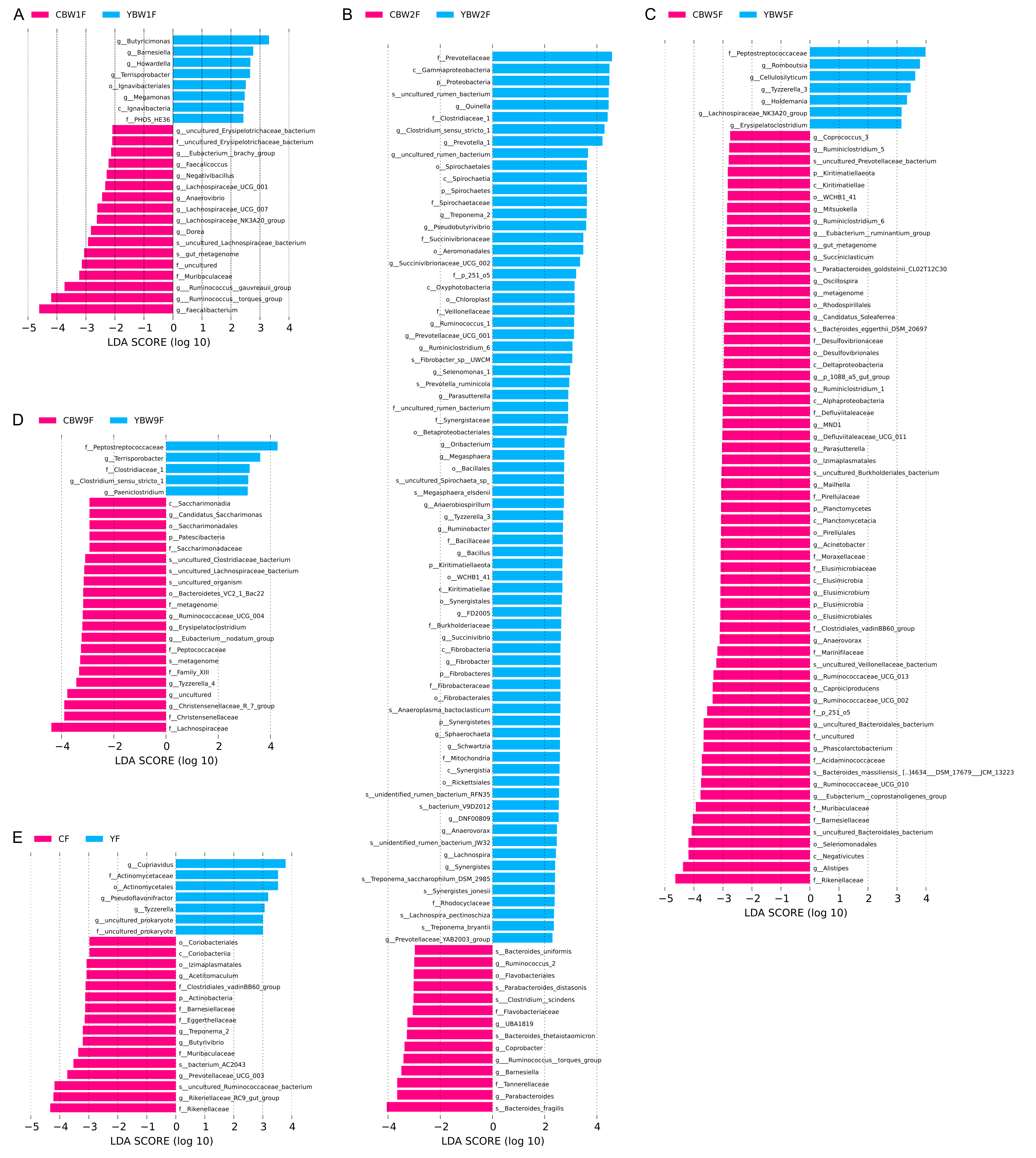


1. Bar plots showing differential abundant fecal microbes of yak and cattle calves at 1 week of age (A), 2 weeks of age (B), 5 weeks of age (C), 9 weeks of age (D), and their mothers at 1 weeks postpartum (E), as identified by linear discriminant analysis (LDA) effect size (LEfSe). The bar plot shows scores for all the taxa with a LDA score ≥ 2. Labels are shown at the family and genera levels.
